# Reflectance Estimation from Multispectral Linescan Acquisitions under Varying Illumination—Application to Outdoor Weed Identification †

**DOI:** 10.3390/s21113601

**Published:** 2021-05-21

**Authors:** Anis Amziane, Olivier Losson, Benjamin Mathon, Aurélien Dumenil, Ludovic Macaire

**Affiliations:** 1 The French National Centre for Scientific Research (CNRS), Lille University, Centrale Lille, UMR 9189 CRIStAL, F-59000 Lille, France; anis.amziane@univ-lille.fr (A.A.); benjamin.mathon@univ-lille.fr (B.M.); ludovic.macaire@univ-lille.fr (L.M.); 2 Chambre d’Agriculture de la Somme, F-80090 Amiens, France; a.dumenil@somme.chambagri.fr

**Keywords:** multispectral imaging, snapscan camera, reflectance estimation, precision farming, crop/weed detection and identification, segmentation, supervised pixel classification

## Abstract

To reduce the amount of herbicides used to eradicate weeds and ensure crop yields, precision spraying can effectively detect and locate weeds in the field thanks to imaging systems. Because weeds are visually similar to crops, color information is not sufficient for effectively detecting them. Multispectral cameras provide radiance images with a high spectral resolution, thus the ability to investigate vegetated surfaces in several narrow spectral bands. Spectral reflectance has to be estimated in order to make weed detection robust against illumination variation. However, this is a challenge when the image is assembled from successive frames that are acquired under varying illumination conditions. In this study, we present an original image formation model that considers illumination variation during radiance image acquisition with a linescan camera. From this model, we deduce a new reflectance estimation method that takes illumination at the frame level into account. We experimentally show that our method is more robust against illumination variation than state-of-the-art methods. We also show that the reflectance features based on our method are more discriminant for outdoor weed detection and identification.

## 1. Introduction

Nowadays, one of the biggest topics in precision farming is to increase yield production while reducing the quantity of chemicals. In order to optimize the application of herbicides in crop fields, less toxic and expensive weed control alternatives can be considered due to recent advances in imaging devices. During the last decade, sophisticated multispectral sensors have been manufactured and deployed in crop fields, leading to weed detection [1,2,3].

Multispectral cameras collect data over a wide spectral range and they provide the ability to investigate the spectral responses of soils and vegetated surfaces in narrow spectral bands. Two main categories of devices can be distinguished in multispectral image acquisition. “Snapshot” (multi-sensor or filter array-based) devices build the image from a single shot [4]. Although this technology provides multispectral images at a video frame rate, the few acquired channels and low spatial resolution may not be sufficient for fully exploring the vegetation spectral signatures. “Multishot” (tunable filter or illumination-based, push-broom, and spatio-spectral linescan) devices build the image from several and successive frame acquisitions [5,6,7]. Despite being restricted to still scenes, they provide images with a high spectral and spatial resolution. We use a multishot camera, called the “Snapscan” to acquire outdoor multispectral radiance images of plant parcels in a greenhouse under skylight [8]. From this radiance information, reflectance is estimated as an illumination-invariant spectral signature of each species. Several methods have been proposed for computing reflectance thanks to prior knowledge regarding cameras or illumination conditions [9,10,11]. In field conditions, the typical methods first estimate the illumination by including a reference device (a white diffuser or a color-checker chart) in the scene [2,12,13,14]. Subsequently, reflectance is estimated at each pixel *p* by the channel-wise division of the value of the radiance image at *p* by the pixel values that characterize the white diffuser or the color-checker white patch. In [15], an extension to the multispectral domain of four algorithms traditionally applied to RGB images is proposed for estimating the illumination. In [16], a Bragg-grating-based multispectral camera acquires outdoor radiance images and reflectance is estimated from two white diffusers, one along the image bottom border and another fully visible one. A coarse reflectance is computed first, and then rescaled using illumination-based scaling factors. Finally, the resulting reflectance image is normalized channel-wise by the average reflectance value that is computed over pixels of the second white diffuser that is present in the scene. In [17], a multispectral camera is used in conjunction with a skyward pointing spectrometer to estimate the reflectance from the acquired scene radiance.

These methods require additional devices and knowledge regarding the spectral sensitivity functions (SSFs) of the sensor filters. They also often assume constant incident illumination throughout a few seconds. However, in outdoor conditions, illumination may vary significantly during the successive frame acquisitions (scans) that last for several seconds.

In this paper, we propose a reflectance estimation method that is robust to illumination variations during the multispectral image acquisition that was performed by the Snapscan camera. In Section 2, we provide details regarding multispectral radiance image acquisition with this device and propose an original model for such image formation. In Section 3, this model is first used to study reflectance estimation under constant illumination. We also show that, when outdoor illumination varies during the frame acquisitions, the assumption about spatio-spectral correlation does not hold. Based on this model, we then propose a new method for estimating the scene reflectance from a multispectral radiance image acquired in uncontrolled and varying illumination conditions (see Section 4). Section 5 presents an experimental evaluation of the proposed reflectance estimation, and Section 6 shows the results of weed/crop segmentation using the estimated reflectance.

## 2. Multispectral Radiance Image Acquisition by Snapscan Camera

In this section, we first detail how the Snapscan camera achieves radiance measurement and frame acquisition. Subsequently, we explain how a multispectral image is obtained from the successively acquired frames. We propose an original multispectral image formation model that handles how illumination is associated to both the considered band and pixel because the radiance that is associated to a given spectral band at a pixel is measured in a frame that is acquired at a specific time. From this new model, we show that the spatio-spectral correlation assumptions do not hold when illumination varies during the frame acquisitions.

### 2.1. Radiance Measurement

The Snapscan is a multispectral camera manufactured by IMEC that embeds a single matrix sensor that is covered by a series of narrow stripes of Fabry-Perot integrated filters. It contains B=192 optical filters whose central wavelengths range from $\lambda^{0}=$ 475.1 nm to $\lambda^{B-1}=$ 901.7 nm with a variable center step (from 0.5 nm to 5 nm). Specifically, each filter of index b∈[0,B−1] is associated with five adjacent rows of 2048 pixels that form a filter stripe, and it samples a band from the visible or near infra-red spectral domain according to its SSF $T^{b}\left(\lambda \right)$ with a full width at half maximum between 2 nm and 10 nm.

**Figure 1:**
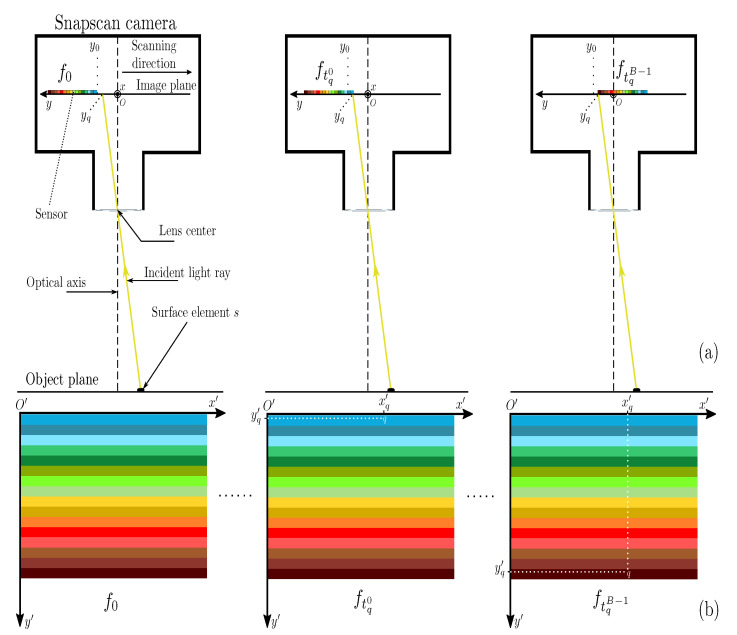
(**a**) Side view of Snapscan camera observing a surface element *s* of a static scene. (**b**) The location of the measured radiance observed at point *q* associated to *s* in frames acquired at $t=0,\;t_{q}^{0},\;t_{q}^{B-1}$.

The Snapscan camera acquires a sequence of frames to provide a multispectral image. During frame acquisitions, the object and camera both remain static while the sensor moves and illumination may change. Therefore, the measurement of the radiance that is reflected by a given lambertian surface element *s* of the scene varies according to the frame acquisition time *t*, although *s* is projected at a fixed point *q* of the image plane. Let us denote, as $E_{t}\left(\lambda \right)$∈[0,1], the relative spectral power distribution (RSPD) of the illumination at *t* and assume that it homogeneously illuminates all of the surface elements of the scene. The radiance that is reflected by *s* and refracted by the camera lens projects onto the image plane at *q* as a stimulus $L_{t,q}\left(\lambda \right)$:

(1) $$L_{t,q}\left(\lambda \right)=E_{t}\left(\lambda \right)\;\cdot \;R_{q}\left(\lambda \right)\;\cdot \;A_{q}\left(\lambda \right),$$

where $R_{q}\left(\lambda \right)$∈[0,1] is the spectral reflectance of the surface element *s* that is observed by *q*, and $A_{q}\left(\lambda \right)$∈[0,1] is the optical attenuation of the camera lens at *q*. All of these functions depend on the wavelength λ. The sensor moves forward on the image plane according to the direction perpendicular to the filter stripes (see Figure 1a). Between two successive frame acquisitions, it moves by a constant step v=5 (in pixels) that is equal to the number of rows in each stripe. Therefore, the radiance that is measured at *q* is filtered by a different Fabry–Perot filter of index $b_{t,q}$ ($0\leq b_{t,q}<B$) at each acquisition time *t*. The radiance at *q* is fully sampled over *N* frame acquisitions, provided that each of them measures the radiance there, i.e., N≥B. Let the coordinates of point *q* be ${\left(x_{q},y_{q}\right)}_{C}$ in the camera 2D coordinate system (O,x,y) whose origin *O* corresponds to the intersection between the optical axis and image plane. The unit vectors of *x* and *y* are given by the photo-sensitive element size (i.e., axis units match with pixels), and *y* is oriented opposite to the sensor movement. At a given point *q*, the filter index $b_{t,q}$ can then be expressed as:

(2) $$b_{t,q}=t+\left\lfloor \frac{y_{q}-y_{0}}{v}\right\rfloor ,$$

where $y_{0}$ is the coordinate along *y* of the first filter row at first acquisition time t=0. Note that the light stimulus $L_{t,q}$ is only associated to a filter at a given point *q* when $t_{q}^{0}\leq t<t_{q}^{B}$. The lower bound $t_{q}^{0}=\left\lfloor (y_{0}-y_{q})/v\right\rfloor$ is the acquisition time at which the first optical filter of the sensor observes $L_{t,q}$. The upper bound $t_{q}^{B}=\left\lfloor (y_{0}-y_{q})/v\right\rfloor +B$ is the time at which all of the sensor filters have observed $L_{t,q}$.

Besides, at a given time *t*, the coordinate $y_{q}$ of point *q* that is associated to a photo-sensitive element of the sensor satisfies:

(3) $$y_{0}-t\cdot v\leq y_{q}<y_{0}+\left(B-t\right)\cdot v$$

since $0\leq b_{t,q}<B$. Given these restrictions, the radiance $S_{t,q}$ that is then measured at *q* by the sensor at acquisition time *t* is expressed as:

(4) $$S_{t,q}=Q\left(\tau \int_{\Omega }L_{t,q}\left(\lambda \right)\;\cdot \;T^{b_{t,q}}\left(\lambda \right)\;d\lambda \right),$$

where *Q* is the quantization function according to the camera bit depth, τ is the integration time of the frames, and Ω is the working spectral domain. Note that τ is set to the highest possible value that provides no saturated pixel.

### 2.2. Frame Acquisition

The radiance that is measured at *q* is stored by the camera as a pixel value $f_{t,q}=S_{t,q}$ in frame $f_{t}$ (see Figure 1b). We define the coordinate system $\left(O^{\prime },x^{\prime },y^{\prime }\right)$ attached to the sensor, such that origin $O^{\prime }$ is the first (top-left) photo-sensitive element location, axis $y^{\prime }$ corresponds to *y*, and $x^{\prime }$ is parallel to *x*, in order to compute the coordinates of *q* relative to the frame. In this frame system, the coordinates of *q* are ${\left(x_{q}^{\prime },y_{q}^{\prime }\right)}_{F}={\left(x_{q},y_{q}-y_{0}+t\;\cdot \;v\right)}_{F}$. Note that Equation (3) allows us to check that $0\leq y_{q}^{\prime }<B\;\cdot \;v$.

Conversely, any given pixel $p{\left(x_{p}^{\prime },y_{p}^{\prime }\right)}_{F}$ of a frame $f_{t}$ is mapped to the coordinates in the camera coordinate system as:

(5) $${\left(x_{p},y_{p}\right)}_{C}={\left(x_{p}^{\prime },y_{p}^{\prime }+y_{0}-t\;\cdot \;v\right)}_{C},$$

at which the stimulus $L_{t,p}\left(\lambda \right)$ of a surface element radiance is filtered by the filter of index $b_{p}=\left\lfloor y_{p}^{\prime }/v\right\rfloor$. From this point of view, each frame pixel value is, therefore, also expressed as:

(6) $$f_{t,p}=S_{t,p}=Q\left(\tau \int_{\Omega }L_{t,p}\left(\lambda \right)\;\cdot \;T^{b_{p}}\left(\lambda \right)\;d\lambda \right).$$

Before the frame acquisitions, the Snapscan uses its internal shutter to acquire a dark frame $f_{dark}$ whose values are subtracted pixel-wise from the acquired frames. Therefore, we assume that the pixel value that is expressed by Equation (6) is free from thermal noise.

Let us also point out that, at two (e.g., successive) acquisition times $t_{1}$ and $t_{2}$, the sensor is at different locations. Therefore it acquires the values $f_{t_{1},p}$ and $f_{t_{2},p}$ from the stimuli $L_{t_{1},p}$ and $L_{t_{2},p}$ of two different surface elements at a given pixel *p* whose coordinate $y_{p}$ in the camera system is time-dependent (see Equation (5)). Besides, the stimuli $L_{t_{1},p}$ and $L_{t_{2},p}$ are filtered by the same filter whose index only depends on the pixel coordinate $y_{p}^{\prime }$ in the frame system. Equations (4) and (6) model the radiance that is measured at a given point in the image plane and stored at a given pixel of a frame, respectively. Both of the equations take account of illumination variation during the frame sequence acquisition, but differently take the sensor movement into account. Indeed, the filter index changes at a given point of the image plane during the frame acquisition (see Equation (4)), whereas the observed surface element changes at a given pixel in the successive frames (see Equation (6)).

### 2.3. Stripe Assembly

**Figure 2:**
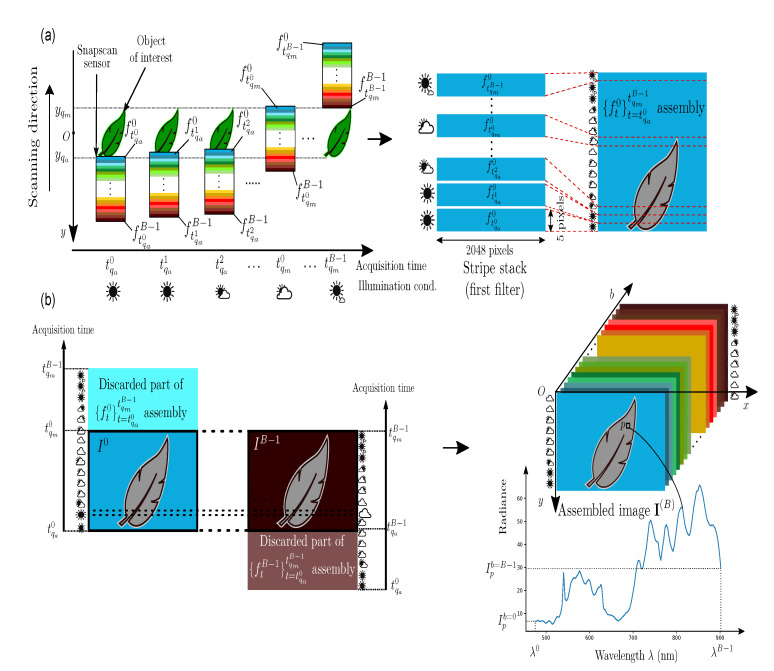
Frame acquisition and stripe assembly for channel $I^{0}$ (**a**) and multispectral image with *B* spectral channels (**b**).

We now determine the first and last acquisition times of the frame sequence that is required to capture an object of interest whose projection points on the image plane are bounded along the *y* axis by $q_{a}{\left(x_{q_{a}},y_{q_{a}}\right)}_{C}$ and $q_{m}{\left(x_{q_{m}},y_{q_{m}}\right)}_{C}$, with $y_{q_{a}}>y_{q_{m}}$. Given the initial coordinate $y_{0}$ of the sensor along *y*, we can compute the first and last frame acquisition times $t_{q_{a}}^{0}$ and $t_{q_{m}}^{B-1}$, so that the measured radiances at the points between $q_{a}$ and $q_{m}$ are consecutively filtered by the *B* sensor filters (see the top part of Figure 2). The acquisition of the multispectral image from the frame sequence ${\left\{f_{t}\right\}}_{t=t_{q_{a}}^{0}}^{t_{q_{m}}^{B-1}}$ takes account of the spatial and spectral organizations of each frame. A frame $f_{t}$ is spatially organized as juxtaposed stripes of *v* adjacent pixel rows. A stripe $f_{t}^{b}$, b=0,⋯,B−1, of *v* adjacent pixel rows contains the spectral information of the scene radiance that is filtered according to the SSF $T^{b}\left(\lambda \right)$ of filter *b* centered at wavelength $\lambda^{b}$. All of the stripes that are associated with filter *b* in the acquired frames are stacked by the assembly function ⨁ to provide a stripe assembly defined as:

(7) $${\left\{f_{t}^{b}\right\}}_{t=t_{q_{a}}^{0}}^{t_{q_{m}}^{B-1}}\overset{\mathrm{def}}{=}⨁\left({\left\{f_{t}\right\}}_{t=t_{q_{a}}^{0}}^{t_{q_{m}}^{B-1}},b\right)={\left[f_{t_{q_{m}}^{B-1}}^{b},\cdots ,f_{t+1}^{b},f_{t}^{b},f_{t-1}^{b},\cdots ,f_{t_{q_{a}^{0}}}^{b}\right]}^{⊺}.$$

The size of each stripe assembly is 2048 pixels in width and N·v pixels in height, where $N=\left\lfloor \left(t_{q_{m}}^{B-1}-t_{q_{a}}^{0}\right)/\Delta \right\rfloor +1$ is the number of acquired frames and Δ is the frame acquisition period.

To form the multispectral image $I^{\left(B\right)}={\left\{I^{b}\right\}}_{b=0}^{B-1}$ of the object of interest, only the scene part that is common to all stripe assemblies is considered by the camera (see the bottom part of Figure 2). Specifically, the retained stripes in the *b*-th assembly are acquired between $t_{q_{a}}^{b}$ and $t_{q_{m}}^{b}$ to form each channel $I^{b}$:

(8) $$I^{b}={\left\{f_{t}^{b}\right\}}_{t=t_{q_{a}}^{b}}^{t_{q_{m}}^{b}}.$$

The multispectral image $I^{\left(B\right)}$ has its own coordinate system. For convenience, in the sequel, we denote a pixel as p(x,y) in this system, since the camera and frame coordinate systems are not used any longer.

### 2.4. Formation Model of a Multispectral Image Acquired by Snapscan Camera

We can now infer an image formation model for multishot linescan cameras, such as the Snapscan. At any pixel *p*, the radiance value $I_{p}^{b}$ that is associated to a channel index b∈[0,B−1] is acquired at $t=t_{p}^{b}$, with $t_{q_{a}}^{b}\leq t_{p}^{b}\leq t_{q_{m}}^{b}$ (see Equation (8)). It results from the light stimulus $L_{t_{p}^{b},p}$ that was filtered according to $T^{b}$ (whose index dependence upon *p* is dropped by stripe assembly step), and is therefore defined from Equations (1) and (6), as:

(9) $$I_{p}^{b}=Q\left(\tau \int_{\Omega }E_{t_{p}^{b}}\left(\lambda \right)\;\cdot \;R_{p}\left(\lambda \right)\;\cdot \;A_{p}\left(\lambda \right)\;\cdot \;T^{b}\left(\lambda \right)\;d\lambda \right).$$

The term $E_{t_{p}^{b}}\left(\lambda \right)$ shown in Equation (9) points out that illumination is associated to both a channel index and a pixel. These dependencies may weaken the spatio-spectral correlation assumptions of the measured scene radiance.

Spectral correlation relies on the assumption that the SSFs that are associated to adjacent spectral channels strongly overlap. Thus, radiance measures at a given pixel in these channels should be very similar (or correlated). Let us consider the radiance values in two channels $b_{1}$ and $b_{2}$ at a given pixel *p*. Even if the SSFs $T^{b_{1}}\left(\lambda \right)$ and $T^{b_{2}}\left(\lambda \right)$ strongly overlap (and are equal in the extreme case), the illumination conditions at $t_{p}^{b_{1}}$ and $t_{p}^{b_{2}}$ are different, hence $I_{p}^{b_{1}}\neq I_{p}^{b_{2}}$.

Spatial correlation relies on the assumption that the reflectance across locally close surface elements of a scene does (almost) not change. Thus, under the same illumination, the radiance measures at their associated pixels within a channel are correlated. Let us consider two pixels, $p_{1}\left(x_{p_{1}},y_{p_{1}}\right)$ and $p_{2}\left(x_{p_{2}},y_{p_{2}}\right)$, which observe surface elements of a scene with the same reflectance $R_{p_{1}}\left(\lambda \right)=R_{p_{2}}\left(\lambda \right)$ for all λ∈Ω. If $|y_{p_{1}}-y_{p_{2}}|\geq v$, then the radiances at $p_{1}$ and $p_{2}$ are acquired at different times $t_{p_{1}}^{b}$ and $t_{p_{2}}^{b}$ associated to different illumination conditions $E_{t_{p1}^{b}}$ and $E_{t_{p2}^{b}}$, hence $I_{p_{1}}^{b}\neq I_{p_{2}}^{b}$.

Therefore, the spatio-spectral correlation assumption does not hold in the image formation model of the Snapscan camera when illumination varies.

## 3. Reflectance Estimation with a White Diffuser under Constant Illumination

This short section introduces how to estimate reflectance by a classical (white diffuser-based) method and how the result should be post-processed to ensure its consistency.

### 3.1. Reflectance Estimation

In order to estimate spectral reflectance from radiance images that were acquired under an illumination that is almost constant over time, one classically uses the image $I^{\left(B\right)}\left[\mathrm{WD}\right]$ of a white diffuser acquired in full field beforehand and assumes that:

(*i*) The illumination is spatially uniform and it does not vary during the frame acquisitions, thus $E_{t_{p}^{b}}\left(\lambda \right)=E\left(\lambda \right)$ for all b∈[0,B−1] and $p\in I^{\left(B\right)}$, and Equation (9) becomes:
  (10) $$I_{p}^{b}=\tau \;\cdot \;\sum_{l=0}^{B-1}E\left(\lambda^{l}\right)\;\cdot \;R_{p}\left(\lambda^{l}\right)\;\cdot \;A_{p}\left(\lambda^{l}\right)\;\cdot \;T^{b}\left(\lambda^{l}\right).$$
  Note that the quantization function *Q* is omitted here, since the different terms are considered as being already quantized.
(*ii*) Each of the Fabry-Perot filters has an ideal SSF $T^{b}\left(\lambda \right)=\delta \left(\lambda -\lambda^{b}\right)=\left\{\begin{array}{cc} 1 & \mathrm{if}\;\lambda =\lambda^{b},\\ 0 & \mathrm{otherwise}, \end{array}\right.$ such that Equation (10) becomes:
  (11) $$I_{p}^{b}=\tau \;\cdot \;E\left(\lambda^{b}\right)\;\cdot \;R_{p}\left(\lambda^{b}\right)\;\cdot \;A_{p}\left(\lambda^{b}\right).$$

Reflectance is then derived for any pixel *p* that is associated to a spectral band centered at $\lambda^{b}$ as:

(12) $$R_{p}\left(\lambda^{b}\right)=\frac{I_{p}^{b}}{\tau \;\cdot \;E\left(\lambda^{b}\right)\;\cdot \;A_{p}\left(\lambda^{b}\right)}.$$

The white diffuser is supposed to be perfectly diffuse and reflect the incident light with a constant diffuse reflection factor $\rho_{\mathrm{wd}}$. Hence, for $I^{\left(B\right)}\left[\mathrm{WD}\right]$, we can write:

(13) $$\rho_{\mathrm{wd}}=\frac{I_{p}^{b}\left[\mathrm{WD}\right]}{\tau_{\mathrm{wd}}\;\cdot \;E\left(\lambda^{b}\right)\;\cdot \;A_{p}\left(\lambda^{b}\right)},$$

where $\tau_{\mathrm{wd}}$ is the frame integration time of $I^{\left(B\right)}\left[\mathrm{WD}\right]$. Plugging Equation (13) into (12) yields the reflectance image that is estimated from a *B*-channel radiance image $I^{\left(B\right)}$:

(14) $${\hat{R}}_{p}^{b}=\rho_{\mathrm{wd}}\;\cdot \;\frac{I_{p}^{b}}{I_{p}^{b}\left[\mathrm{WD}\right]}\;\cdot \;\frac{\tau_{\mathrm{wd}}}{\tau }.$$

This reflectance estimation model implicitly compensates the vignetting effect, since the white diffuser and object (scene of interest) occupy the same (full) field of view. Accordingly, $I_{p}^{b}$ and $I_{p}^{b}\left[\mathrm{WD}\right]$ are affected by the same optical attenuation whose effect vanishes after division.

The estimated *B*-channel reflectance image ${\hat{R}}^{\left(B\right)}$ should then undergo two post-processing steps: spectral correction and negative value removal.

### 3.2. Spectral Correction

Each of the Snapscan Fabry–Perot filters is designed to sample a specific spectral band from the spectrum according to its SSF $T^{b}\left(\lambda \right)$. However, because of the SSFs and optical properties of some filters (angular dependence [18], high-energy harmonics), several spectral bands are redundant, which limits the accuracy of the spectral imaging system. This leads to redundancy in spectral bands and introduces spectral information bias. Therefore, the reflectance image with B=192 spectral channels is spectrally corrected and only K=141 channels are kept in practice.

The spectral correction of ${\hat{R}}^{\left(B\right)}$ provides a spectrally corrected *K*-channel reflectance image ${\hat{R}}^{\left(K\right)}$ that is expressed at each pixel *p* as:

(15) $${\hat{R}}_{p}^{\left(K\right)}=M\;\cdot \;{\hat{R}}_{p}^{\left(B\right)},$$

where M is the sparse K×B correction matrix that is provided by the calibration file of our Snapscan camera. The linear combinations of the channel values of ${\hat{R}}^{\left(B\right)}$ according to Equation (15) are designed by the manufacturer to remove the redundant channels and attenuate second-order harmonics. This spectral correction provides new centers ${\left\{\lambda^{k}\right\}}_{k=0}^{K-1}$ for the bands (referred to as “virtual” bands by IMEC) that are associated to the image channels, but the spectral working domain Ω=[475.1nm,901.7nm] is unchanged.

### 3.3. Negative Value Removal

The acquired radiance image contains negative values due to dark frame subtraction, when the value of a dark frame pixel is higher than the measured radiance at this pixel. This generally occurs in low-dynamics channels, where the central wavelengths are in the range [475.1 nm, 560.4 nm] (before spectral correction). These negative values may lay on vegetation pixels and corrupt reflectance estimation at these pixels. Because we intend to classify vegetation pixels, this could lead to unexpected prediction errors. Negative values also occur—for even more pixels—in the spectrally-corrected reflectance image ${\hat{R}}^{\left(K\right)}$ (see Equation (15)), because the correction matrix *M* contains negative coefficients.

Negative values have no physical meaning and they must be discarded. Because our images mostly contain smooth textures (vegetation, reference panels, soil), we consider that, unlike radiance, reflectance values are highly correlated over close surface elements. Thus, we propose correcting negative values in image ${\hat{R}}^{\left(K\right)}$ by conditionally using a 3×3 median filter, as:

(16) $${\hat{R}}_{\mathrm{ref},p}^{k}=\left\{\begin{array}{cc} \underset{3\times 3}{\mathrm{median}}\left\{{\hat{R}}_{p}^{k}\right\} & \mathrm{if}\;{\hat{R}}_{p}^{k}<0,\\ {\hat{R}}_{p}^{k} & \mathrm{otherwise}, \end{array}\right.$$

where ${\hat{R}}_{\mathrm{ref},p}^{k}$ is the final reflectance value at pixel *p* for channel *k*. Because we consider the reflectance that is estimated by this model (Equations (14)–(16)) as a reference, it is denoted as ${\hat{R}}_{\mathrm{ref}}^{\left(K\right)}$.

## 4. Outdoor Reflectance Estimation with Reference Devices in the Scene

Because illumination varies during the acquisitions of outdoor scene images, the reflectance estimation method that is described by Equation (14) is not adapted to linescan cameras, such as the Snapscan. In such a case, one solution is to use several reference devices [16].

**Figure 3:**
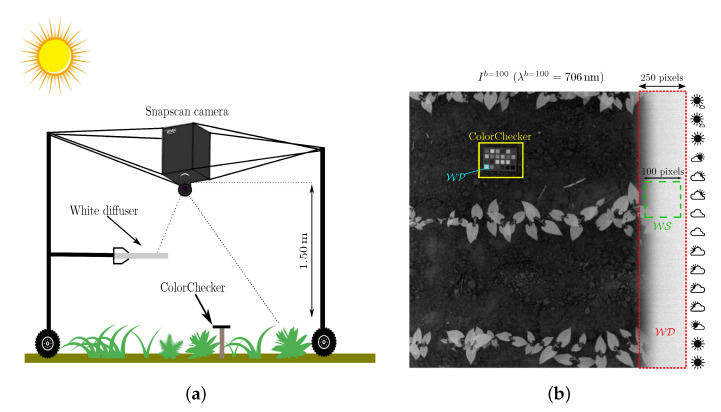
(**a**) The acquisition setup with the camera mounted on its top. (**b**) Channel of a radiance image with the ColorChecker and a white diffuser along its right border. Pixel subsets WD, WS, and WP are displayed in red, green, and cyan, respectively.

As a first reference device, we use a white diffuser tile mounted on the acquisition system, so that the sensor vertically observes a portion of it (see Figure 3a). Therefore, the pixel subset WD contains (about 10%) right border pixels that represent the white diffuser, as shown in Figure 3b. Because WD spans all the image rows, we further extract a small white square WS that represents a sample of this reference device. Each acquired image also contains a GretagMacbeth${}^{™}$ ColorChecker that is principally used to assess the performances that are reached by reflectance estimation methods. The pixel subset WP representing the ColorChecker white patch is used as a second reference device by the double white diffuser (dwd) method [16] that we have adapted to our Snapscan acquisitions, as described in Appendix A.

Although the vignetting effect only depends on the intrinsic camera properties, this method corrects it in each acquired image. In Section 4.1, we propose performing this correction by the analysis of the white diffuser image $I^{\left(B\right)}\left[\mathrm{WD}\right]$. Subsequently, we present state-of-the art estimation reflectance methods that only require one reference device in the scene, but assume that the illumination is constant during the frame acquisition. We finally propose a single-reference method to estimate reflectance in the case of varying illumination.

### 4.1. Vignetting Correction

The dwd method acquires a full-field white diffuser image before each scene image acquisition in order to correct the vignetting effect [16]. However, this procedure can be cumbersome, since it requires an external intervention in order to place/remove the full-field white diffuser. Other methods that are presented in the following only require correcting it only once. Because we consider that vignetting only depends on the intrinsic geometric properties of the camera, we propose correcting it thanks to the analysis of a single full-field white diffuser image $I^{\left(B\right)}\left[\mathrm{WD}\right]$ acquired in a laboratory under controlled illumination conditions.

The vignetting effect refers to a loss in the intensity values from the image center to its borders due to the geometry of the sensor optics. To highlight how the vignetting effect would affect radiance measurements, let us rewrite Equation (9) under the Dirac SSF assumption as:

(17) $$I_{p}^{b}=\tau \;\cdot \;E_{t_{p}^{b}}\left(\lambda^{b}\right)\;\cdot \;R_{p}\left(\lambda^{b}\right)\;\cdot \;A_{p}\left(\lambda^{b}\right).$$

In order to compensate for the spatial variation of $A_{p}\left(\lambda^{b}\right)$, we compute a correction factor at *p*, because it requires no knowledge regarding the optical device behavior [19]. Being deduced from the full-field white diffuser image $I^{\left(B\right)}\left[\mathrm{WD}\right]$, the correction factor is channel-wise and pixel-wise computed as:

(18) $$C_{p}^{b}=\frac{{\underline{I}}^{b}\left[\mathrm{WD}\right]}{I_{p}^{b}\left[\mathrm{WD}\right]},$$

where ${\underline{I}}^{b}\left[\mathrm{WD}\right]$ is the median value of the *m* pixels (m=11 in our experiments) with the highest values over $I_{p}^{b}\left[\mathrm{WD}\right]$, which discards saturated or defective pixel values. The correction factors are stored in a *B*-channel multispectral image, denoted as C.

Because C is deduced from a single white diffuser image, it would be corrupted by noise (even after thermal noise removal during the frame acquisitions). Thus, we propose to directly denoise C by convolving each of its channels $C^{b}$ with an 11×11 averaging filter H:

(19) $${\tilde{C}}^{b}=H*C^{b}.$$

The vignetting effect in the *B*-channel radiance image $I^{\left(B\right)}$ is corrected channel-wise and pixel-wise using the smoothed correction factors:

(20) $${\tilde{I}}_{p}^{b}={\tilde{C}}_{p}^{b}\;\cdot \;I_{p}^{b},$$

where $I_{p}^{b}$ and ${\tilde{I}}_{p}^{b}$ are the intensity values before and after vignetting correction. This procedure should reduce noise while preserving image textures. We assume that the attenuation is spatially uniform after vignetting correction (i.e., $A_{p}\left(\lambda^{b}\right)\;\cdot \;{\tilde{C}}_{p}^{b}=\alpha^{b}\in R$ for any given channel index *b* and pixel *p*), such that each value of the vignetting-free radiance image is expressed from Equation (17) as:

(21) $${\tilde{I}}_{p}^{b}=\tau \;\cdot \;R_{p}\left(\lambda^{b}\right)\;\cdot \;E_{t_{p}^{b}}\left(\lambda^{b}\right)\;\cdot \;\alpha^{b}.$$

### 4.2. Reflectance Estimation with One Reference Device under Constant Illumination

The illumination $E_{t_{p}^{b}}\left(\lambda^{b}\right)$ that is associated to *p* can be determined using the radiance measured at a white diffuser pixel $p_{WD}\in \mathrm{WD}$. To determine illumination thanks to a single white diffuser as reference device, the methods in the literature often assume that illumination is constant, i.e., $E_{t_{p}^{b}}\left(\lambda^{b}\right)=E_{t_{p_{WD}}^{b}}\left(\lambda^{b}\right)=E\left(\lambda^{b}\right)$. Equation (21) then becomes ${\tilde{I}}_{p}^{b}=\tau \;\cdot \;R_{p}\left(\lambda^{b}\right)\;\cdot \;E\left(\lambda^{b}\right)\;\cdot \;\alpha^{b}$ and, specifically, ${\tilde{I}}_{p_{WD}}^{b}=\tau \;\cdot \;\rho_{\mathrm{wd}}\;\cdot \;E\left(\lambda^{b}\right)\;\cdot \;\alpha^{b}$, since the white diffuser has a homogeneous diffuse reflection $R_{p_{WD}}\left(\lambda^{b}\right)=\rho_{\mathrm{wd}}$ (95% in our case). The reflectance at *p* is then deduced from ${\tilde{I}}_{p}^{b}$ and ${\tilde{I}}_{p_{WD}}^{b}$ as:

(22) $$R_{p}\left(\lambda^{b}\right)=\rho_{\mathrm{wd}}\;\cdot \;\frac{{\tilde{I}}_{p}^{b}}{{\tilde{I}}_{p_{WD}}^{b}}.$$

To be robust against spatial noise, the white-average (wa) method [2,20] averages all of the values over the white diffuser pixel subset WS (see Figure 3b) and estimates the reflectance at each image pixel as:

(23) $${\hat{R}}_{\mathrm{wa},p}^{b}=\rho_{\mathrm{wd}}\;\cdot \;\frac{{\tilde{I}}_{p}^{b}}{\frac{1}{\left|\mathrm{WS}\right|}\sum_{s\in \mathrm{WS}}{\tilde{I}}_{s}^{b}},$$

where |·| is the set cardinal.

Similarly, the max-spectral (ms) method [15] assumes that the pixel with maximum value within each channel can be considered to be a white diffuser pixel for estimating the illumination. While ignoring the diffuse reflection factor, reflectance is estimated at each pixel in each channel by the ms method, as:

(24) $${\hat{R}}_{\mathrm{ms},p}^{b}=\frac{{\tilde{I}}_{p}^{b}}{\max_{s\in X}{\tilde{I}}_{s}^{b}},$$

where *X* contains all of the image pixels, except WD, and those of the ColorChecker.

The wa and ms-based *B*-channel reflectance images undergo spectral correction and negative value removal (see Equations (15) and (16)) to provide the final *K*-channel reflectance images ${\hat{R}}_{\mathrm{wa}}^{\left(K\right)}$ and ${\hat{R}}_{\mathrm{ms}}^{\left(K\right)}$.

### 4.3. Reflectance Estimation with One Reference Device under Varying Illumination

In varying illumination conditions, the Snapscan acquires each row at a given time, hence under a specific illumination (see Section 2.4). Hence, reflectance can no longer be estimated, as in Equation (14). Instead, we propose determining the illumination that is associated to each row of the vignetting-free image ${\tilde{I}}^{\left(B\right)}$ from the white diffuser pixel set WD [21]. The underlying assumption is that illumination is spatially uniform over each row at both the white diffuser and scene pixels (that may be not verified in the case of shadows).

Based on this row uniformity assumption for illumination, we estimate reflectance from ${\tilde{I}}^{\left(B\right)}$ in a row-wise manner, as follows. At pixel *p* with spatial coordinates $x_{p}$ and $y_{p}$, Equation (21) can be rewritten as:

(25) $$R_{p}\left(\lambda^{b}\right)=\frac{{\tilde{I}}_{p}^{b}}{\tau \;\cdot \;E_{t_{y_{p}}^{b}}\left(\lambda^{b}\right)\;\cdot \;\alpha^{b}}.$$

To determine the illumination $E_{t_{y_{p}}^{b}}\left(\lambda^{b}\right)$ that is associated to the row of *p* for channel index *b*, we use a white diffuser pixel $r_{WD}\in \mathrm{WD}$ located on the same row as *p*. At $r_{WD}$, the reflectance is equal to the white diffuser reflection factor $\rho_{\mathrm{wd}}$, and Equation (21) provides the vignetting-free radiance as:

(26) $${\tilde{I}}_{r_{WD}}^{b}=\tau \;\cdot \;\rho_{\mathrm{wd}}\;\cdot \;E_{t_{r_{WD}}^{b}}\left(\lambda^{b}\right)\;\cdot \;\alpha^{b}.$$

Because *p* and $r_{WD}$ are located on the same row, $t_{y_{p}}^{b}=t_{r_{WD}}^{b}$ and $E_{t_{y_{p}}^{b}}\left(\lambda^{b}\right)=E_{t_{r_{WD}}^{b}}\left(\lambda^{b}\right)$ according to the assumption regarding the spatial uniformity over each row. Therefore, Equation (26) can be rewritten as:

(27) $$E_{t_{y_{p}}^{b}}\left(\lambda^{b}\right)=\frac{{\tilde{I}}_{r_{WD}}^{b}}{\tau \;\cdot \;\rho_{\mathrm{wd}}\;\cdot \;\alpha^{b}},$$

which can be considered to be an estimation of the illumination that is associated to pixel *p*. For robustness sake, we propose computing it from the median value ${\tilde{\underline{I}}}_{\mathrm{WD},y_{p}}^{b}$ of the *m* highest pixel values that represent the white diffuser subset WD in $y_{p}$, rather than from a single value ${\tilde{I}}_{r_{WD}}^{b}$. Plugging Equation (27) in (25) yields our row-wise (rw) reflectance estimation at pixel *p* for channel index *b*:

(28) $${\hat{R}}_{\mathrm{rw},p}^{b}=\rho_{\mathrm{wd}}\;\cdot \;\frac{{\tilde{I}}_{p}^{b}}{{\tilde{\underline{I}}}_{\mathrm{WD},y_{p}}^{b}}.$$

In practice, setting m=11 pixels is a good compromise for accurately estimating the illumination for each row and each channel.

## 5. Experiments about Outdoor Reflectance Estimation

We now present the experimental setup and metrics that were used to objectively evaluate the estimated reflectance. The accuracy results are obtained and discussed for the previously described estimation methods as well as for the extra training-based method described in the present section.

### 5.1. Experimental Setup

**Figure 4:**
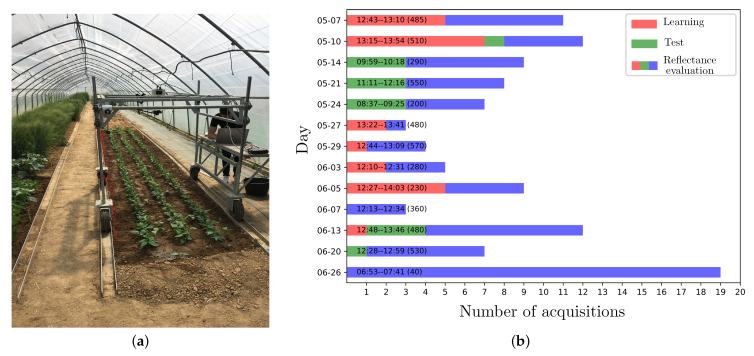
(**a**) Our experimental site and apparatus for vegetation image acquisitions. (**b**) The acquisition dates and times of the 109 images that were provided by our 2019 acquisition campaign. The text along each bar gives the acquisition time range and a coarse estimation of global solar irradiance (W·m${}^{-2}$) at the median acquisition time in parentheses [22]. The images used to assess supervised beet (crop) and weed detection/identification (see Section 6) are shown in red and green, other images in blue. All of the images are used to assess reflectance estimation quality. Series are stacked for readability and their order is not meaningful in regards to any acquisition time order.

An acquisition campaign that was conducted in a greenhouse under skylight (see Figure 4a) provided 109 radiance images of 2048×2048 pixels × 192 channels of 10-bit depth. Among the targeted plants are crops (e.g., beet) and weeds (e.g., thistle and goose-foot). The images were acquired at different dates of May and June 2019, and different day times (see Figure 4b). Figures 6a and 7a show a RGB rendering of two of them with the D65 illuminant.

**Figure 5:**
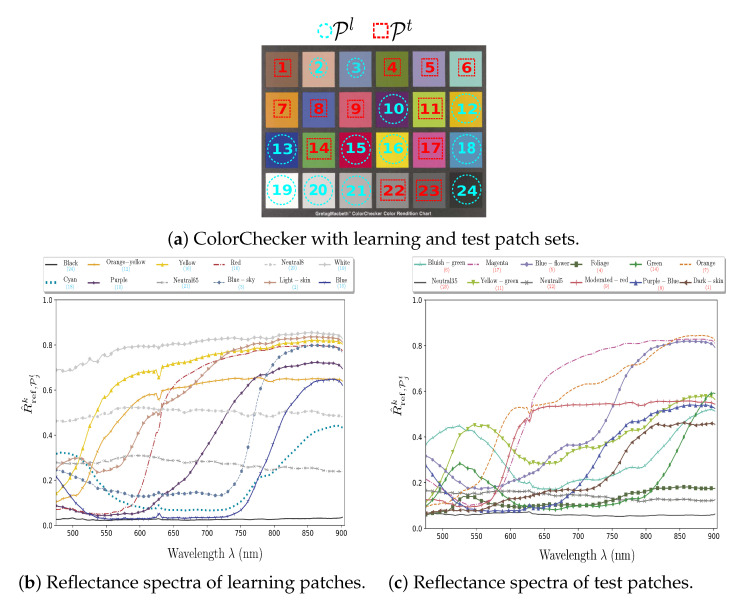
(**a**) ColorChecker patch numbers and (**b**,**c**) reference reflectance spectra.

All of the images contain a GretagMacbeth${}^{™}$ ColorChecker that is composed of 24 patches. From $I^{\left(B\right)}\left[\mathrm{WD}\right]$ and a radiance image $I^{\left(B\right)}\left[\mathrm{CC}\right]$ of our ColorChecker acquired in a laboratory under controlled illumination, we estimate the *K*-channel reference reflectance image ${\hat{R}}_{\mathrm{ref}}^{\left(K\right)}\left[\mathrm{CC}\right]$ of the ColorChecker according to Equations (14)–(16). From ${\hat{R}}_{\mathrm{ref}}^{\left(K\right)}\left[\mathrm{CC}\right]$, we compute the *K*-dimensional reflectance vector (see Figure 5b,c) of each patch $P_{j}^{}$ as:

(29) $${\hat{R}}_{\mathrm{ref},P_{j}}^{k}\left[\mathrm{CC}\right]=\frac{1}{|P_{j}|}\sum_{p\in P_{j}}{\hat{R}}_{\mathrm{ref},p}^{k}\left[\mathrm{CC}\right],$$

where $|P_{j}|$ is the number of pixels that characterize the considered patch.

Among the 24 color patches of the ColorChecker chart, we use a learning subset $P^{l}$ of 12 patches for the learning procedure and the remaining 12 test patches $P^{t}$ for testing the quality of reflectance estimation (see Figure 5). The learning patches of $P^{l}$ are selected using an exhaustive search.

Among the 2,704,156 tested combinations, we retain the one that provides the lowest (mean absolute) reflectance estimation error (see Section 5.4).

The test subset $P^{t}$ is used to assess the performances that are reached by reflectance estimation methods, and the learning one $P^{l}$ is fed into a training-based reflectance estimation method, as described in the following.

### 5.2. Training-Based Reflectance Estimation

The linear Wiener (wn) estimation technique can be applied to estimate reflectance thanks to a learning procedure [23]. It is based on a matrix G that transforms radiance spectra into reflectance. From any radiance image $I^{\left(B\right)}$ in the database, we compute the spectrally-corrected vignetting-free radiance image ${\tilde{I}}^{\left(K\right)}$ while using Equations (15) and (20), and then estimate the *K*-channel reflectance image as:

(30) $${\hat{R}}_{\mathrm{wn},p}^{\left(K\right)}=G\;\cdot \;{\tilde{I}}_{p}^{\left(K\right)}.$$

To compute G, we use the spectra of the ColorChecker learning patches ($P^{l}$ subset) that are represented in each of our images. The estimation matrix **G** that is associated to each input radiance image is determined as:

(31) $$G=T_{ref}\;\cdot \;T_{rad}^{⊺}{\left(T_{rad}\;\cdot \;T_{rad}^{⊺}\right)}^{-1},$$

where $T_{ref}$ and $T_{rad}$ are the K×12 matrices that are formed by horizontally stacking the centered and transposed reference reflectance vectors (from ${\hat{R}}_{\mathrm{ref}}^{\left(K\right)}\left[\mathrm{CC}\right]$) and radiance vectors (from the current image ${\tilde{I}}^{\left(K\right)}$) of the learning patches, and ${}^{⊺}$ denotes the transpose.

### 5.3. Evaluation Metrics

To evaluate the accuracy of reflectance estimation, we use the patches of the ColorChecker test subset $P_{}^{t}$ (see Figure 5). Let ${\hat{R}}_{*,P_{j}^{t}}^{\left(K\right)}$, $*\in \left\{\mathrm{rw},\mathrm{wa},\mathrm{ms},\mathrm{dwd},\mathrm{wn}\right\}$ denote the reflectance image that is estimated for patch $P_{j}^{t}\in P_{}^{t}$ by either the proposed rw method (see Equation (28)) or the four implemented state-of-art methods (see Equations (23)–(30)).

This vector is compared to the reference reflectance ${\hat{R}}_{\mathrm{ref},P_{j}^{t}}^{\left(K\right)}\left[\mathrm{CC}\right]$ of the same patch computed according to Equation (29). The spectra of the ColorChecker patches should be similar (and ideally superposed) to their laboratory counterparts when outdoor reflectance is well estimated.

We objectively assess each estimated reflectance image thanks to the mean absolute error (MAE) and angular error Δθ of each test patch $P_{j}^{t}\in P^{t}$ given by:

(32) $$\mathrm{MAE}\left({\hat{R}}_{\mathrm{ref},P_{j}^{t}}^{\left(K\right)}[\mathrm{CC}],{\hat{R}}_{*,P_{j}^{t}}^{\left(K\right)}\right)=\frac{1}{K}\sum_{k=1}^{K}\left|{\hat{R}}_{\mathrm{ref},P_{j}^{t}}^{k}\left[\mathrm{CC}\right]-{\hat{R}}_{*,P_{j}^{t}}^{k}\right|,$$

and:

(33) $$\Delta \theta \left({\hat{R}}_{\mathrm{ref},P_{j}^{t}}^{\left(K\right)}[\mathrm{CC}],{\hat{R}}_{*,P_{j}^{t}}^{\left(K\right)}\right)=\arccos\left(\frac{\langle {\hat{R}}_{\mathrm{ref},P_{j}^{t}}^{k}[\mathrm{CC}],{\hat{R}}_{*,P_{j}^{t}}^{k}\rangle }{{∥{\hat{R}}_{\mathrm{ref},P_{j}^{t}}^{\left(K\right)}\left[\mathrm{CC}\right]∥}_{2}\;\cdot \;{∥{\hat{R}}_{*,P_{j}^{t}}^{\left(K\right)}∥}_{2}}\right),$$

where ${∥\;\cdot \;∥}_{2}$ is the Euclidean norm. When Δθ between two vectors (spectra in our case) is equal to zero, it means that these two vectors are collinear.

### 5.4. Results

We compute the mean absolute error ${\mathrm{MAE}}_{*}$ and angular error ${\Delta \theta }_{*}$ averaged over all of the test patches of all reflectance images estimated from the whole database to obtain aggregated metrics. Table 1 presents the results for the five tested methods.

The MAE and Δθ are complementary metrics and they, respectively, highlight two important properties: the scale and shape of the estimated spectra. Indeed, while MAE is mainly sensitive to the scale of the estimated spectra, Δθ especially focuses on the shape of the spectra, because it is a scale-insensitive measure. Consequently, there might be no correlation between the results that were obtained by the MAE measure and those obtained by Δθ.

No method provides the best results according to the two metrics, as we can see from Table 1. Indeed, the wn and dwd methods provide better results than rw and wa according to the MAE, but the rw and wa methods provide better results in terms of Δθ.

The ms method provides the worst results, because it only analyzes pixels of background and vegetation that strongly absorb the incident light in the visible domain. Hence, the biased illumination estimation in this domain affects the performance of ms method. It is worthwhile to mention that the wn method performance might also be biased, since it uses some of the ColorChecker patches as training references (to build estimation matrix G), while the other patches of the same chart are used to evaluate the reflectance estimation quality.

Among illumination-based methods that analyze a single reference device, rw provides similar results to wa in terms of Δθ, as well as better MAE results. This shows that taking account of the illumination variation during the frame acquisitions improves the reflectance estimation quality.

## 6. Multispectral Image Segmentation

Now, we evaluate the contribution of our proposed rw-based reflectance estimation method for supervised crop/weed detection and identification. For this experiment, we focus on the beet (crop) that must be distinguished from thistle and goose-foot (weeds). First, vegetation pixels are detected and ground truth (labels) regarding vegetation pixels is provided by an expert in agronomy (Section 6.1). In order to evaluate the robustness of each considered feature against illumination conditions, we use a data set composed of 37 radiance (13 single-species and 24 mixed) images that we split into a learning and test set, denoted as $S^{learn}$ (23 images) and $S^{test}$ (14 images) (Section 6.2). The illumination conditions are various in the two sets and $S^{test}$ mostly includes images that are acquired on different days from those of $S^{learn}$ (see Figure 4). Note that, as a consequence, vegetation in the learning and test image sets may not be exactly at the same growth stages. We first compare the discrimination power of reflectance features provided by our rw method against radiance features to assess each reflectance estimation method for crop/weed identification and detection. Subsequently, we compare it with reflectance features that are estimated using each of the four considered state-of-the-art methods (wa,ms,wn, and dwd) (Section 6.3, Section 6.4 and Section 6.5).

### 6.1. Vegetation Pixel Extraction and Labelling

Only vegetation pixels are analyzed because we aim to detect/identify crops and weeds. They are distinguished from the background (white diffuser, ColorChecker, and soil pixels) using the normalized difference vegetation index (NDVI) [24]. We compute the NDVI values from the rw-based reflectance image ${\hat{R}}_{\mathrm{rw}}^{\left(K\right)}$, since the rw method considers illumination variation, but the images provided by any other reflectance estimation method should yield similar vegetation pixel detection results. We consider *p* to be a vegetation pixel if its NVDI value is greater than a threshold γ:

(34) $$\frac{{\hat{R}}_{\mathrm{rw},p}^{139}-{\hat{R}}_{\mathrm{rw},p}^{67}}{{\hat{R}}_{\mathrm{rw},p}^{139}+{\hat{R}}_{\mathrm{rw},p}^{67}}\geq \gamma ,$$

with the Snapscan “virtual” band centers $\lambda^{67}=678.2\;\mathrm{nm}$ and $\lambda^{139}=899.2\;\mathrm{nm}$. Setting γ=0.45 experimentally provides a good compromise between under- and over-segmentation of vegetation pixels. Noisy vegetation pixels are filtered out as much as possible by morphological opening. The vegetation pixels are then manually labelled by an expert in agronomy to build the segmentation ground truth for each multispectral image.

### 6.2. Learning and Test Vegetation Pixels

From the learning set $S^{learn}$, we randomly extract N learning pixels per class. For a given class $C_{i}$, $i=[0,\cdots ,N_{C}-1]$, the number of extracted learning pixels per image depends on the number of images where class $C_{i}$ is represented in $S^{learn}$ (occurrences). Among the 23 learning images, the beet (crop) class appears in 17 images, thistle in nine images, and goosefoot in 12 images.

In the test set $S^{test}$, beet, thistle, and goosefoot are represented, respectively, in 12, 10, and four images. For the weed detection task, we extract 2N learning pixels, half for crop and half for weed class. Because we merge thistle and goosefoot prototype pixels to build a single weed class, we extract N/2 learning pixels for thistle and N/2 for goosefoot.

Each pixel is characterized by a *K*-dimensional (K=141) feature vector of reflectance (or radiance) values. The reflectance/radiance images are averaged channel-wise over a 5×5 pixel window to reduce noise and within-class variability. Table 2 shows the number of learning and test pixels per class for weed detection and beet/thistle/goosefoot identification. All of the available pixels in $S^{test}$ are used to assess the generalization power of a supervised classifier.

### 6.3. Evaluation Metrics

The classical accuracy score can be a misleading measure to evaluate a classifier performance when the number of test pixels that are associated to each class is highly skewed (like in Table 2) [25] (p. 114). A classification model that predicts the majority class for all test pixels reaches a high classification accuracy. However, this model can also be considered as weak when misclassifying pixels of the minority classes is worse than missing pixels from the majority classes. In order to overcome this so-called “accuracy paradox”, the performance of a classification model for imbalanced datasets should be summarized with appropriate metrics, such as precision/recall curve [25] (pp. 53–56, 114). Although some metrics may be more meaningful and easy to interpret, there is no consensus in the literature for choosing a single optimal metric. In our case, we want to correctly detect weed pixels without over-detection, because this would imply spraying crops with herbicides. Therefore, the performance of our classification model on both crop and weed detection/identification should be comparable. For this purpose, we use the per-class accuracy score and the weighted overall accuracy score. We also compute the F1-score that combines the precision and recall measures. These three measures should summarize the classification performance of imbalanced sets of test pixels well.

Let us denote the true test pixel labels as *y* and the set of predicted labels as $\hat{y}$. The per-class accuracy score for class $C_{i}$, $i=[0,\cdots ,N_{C}-1]$, is:

(35) $${\mathrm{Accuracy}}_{C_{i}}=\frac{1}{|y_{C_{i}}|}\sum_{j=0}^{|y_{C_{i}}|-1}\delta \left({\hat{y}}_{C_{ij}}=y_{C_{ij}}\right),$$

where $y_{C_{ij}}$ and ${\hat{y}}_{C_{ij}}$ are the true and predicted labels for the *j*-th test pixel of class $C_{i}$, respectively, and $|y_{C_{i}}|$ is the number of test pixels of class $C_{i}$.

The weighted overall accuracy for binary and multiclass classifications is defined as:

(36) $$\bar{\mathrm{Accuracy}}=\frac{\sum_{i=0}^{N_{C}-1}\omega_{C_{i}}\;\cdot \;{\mathrm{Accuracy}}_{C_{i}}}{\sum_{i=0}^{N_{C}-1}\omega_{C_{i}}},$$

where $\omega_{C_{i}}=1/\left|y_{C_{i}}\right|$ is the weight that is associated to class $C_{i}$ and computed as the inverse of its size, so as to handle imbalanced classes.

Because the F1-score privileges the classification of true positives pixels (weed pixels in our case), we compute the overall $\bar{F1}$-score as the population-weighted F1-score, so that the performances over all classes are considered:

(37) $$\bar{F1}=\frac{\sum_{i=0}^{N_{C}-1}\omega_{C_{i}}\;\cdot \;F1_{C_{i}}}{\sum_{i=0}^{N_{C}-1}\omega_{C_{i}}}.$$

The F1-score of class $C_{i}$ is computed as:

(38) $$F1_{C_{i}}=2\;\cdot \;\frac{{\mathrm{Precision}}_{C_{i}}\;\cdot \;{\mathrm{Recall}}_{C_{i}}}{{\mathrm{Precision}}_{C_{i}}+{\mathrm{Recall}}_{C_{i}}},$$

where

(39) $${\mathrm{Precision}}_{C_{i}}=\frac{\sum_{j=0}^{|y_{C_{i}}|-1}\delta \left({\hat{y}}_{C_{ij}}=y_{C_{ij}}\right)}{|{\hat{y}}_{C_{i}}|},$$

and

(40) $${\mathrm{Recall}}_{C_{i}}=\frac{\sum_{j=0}^{|y_{C_{i}}|-1}\delta \left({\hat{y}}_{C_{ij}}=y_{C_{ij}}\right)}{|y_{C_{i}}|}.$$

### 6.4. Classification Results

The parametric LightGBM (LGBM) and non-parametric Quadratic Discriminant Analysis (QDA) classifiers are applied for supervised weed detection and identification problems. The choice of these two non-linear classifiers is motivated by their processing time during the learning and prediction procedures and their fundamentally different decision rules. Indeed, LGBM is a parametric tree-based classifier that requires a learning procedure to model a complex classification rule, whereas QDA is a simple non-parametric classifier that is based on Bayes’ theorem to perform predictions. For LGBM, we retain the default parameter values (learning rate of 0.05, 150 leaves) and use the log loss function as the learning evaluation metric. LGBM uses a histogram-based algorithm to bucket the features into discrete bins, which drastically reduces the memory and time consumption. The number of bins is set to 255 and the number of boosting operations to 100. Additionally, the feature fraction and bagging fraction parameters are set to 0.8 to increase LGBM speed and avoid over-fitting.

**Figure 6:**
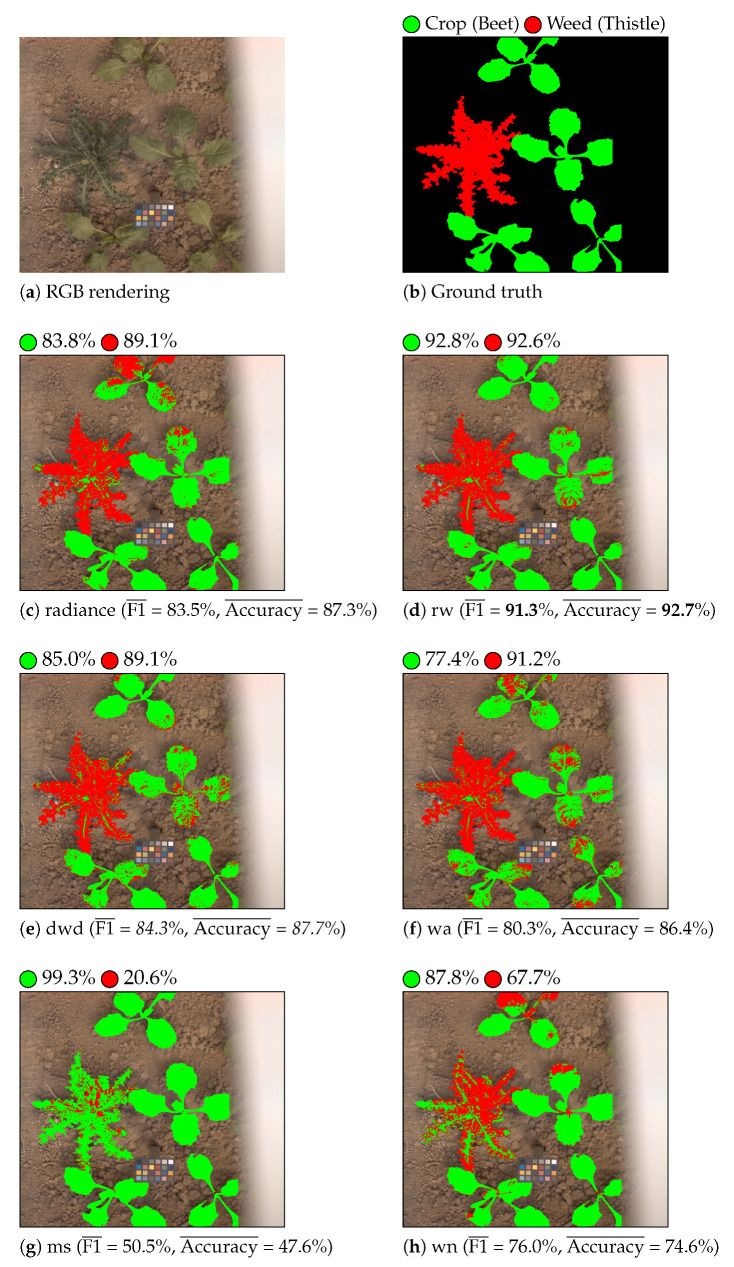
Crop/weed detection. Beet is displayed as green and weed as red. The per-class accuracy score is displayed near each colored circle (class label) for each considered feature.

**Figure 7:**
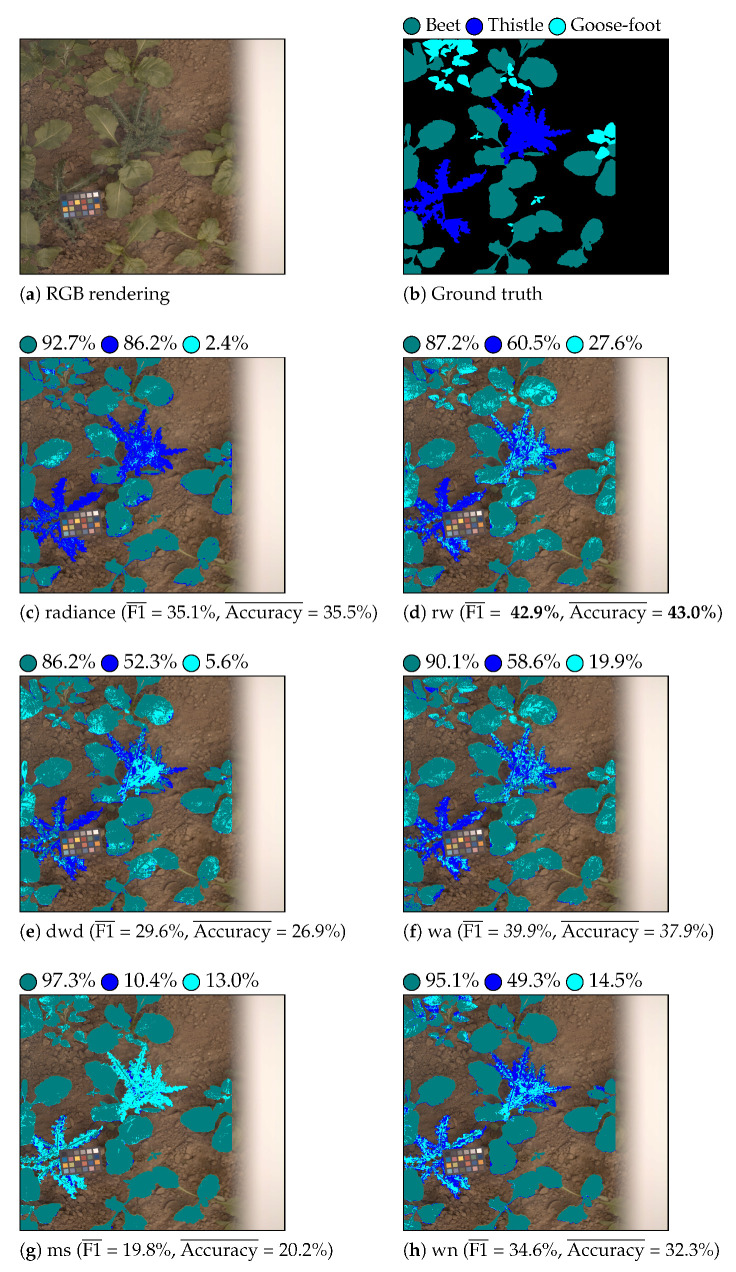
Beet/thistle/goosefoot identification. Beet is displayed as teal, thistle as blue, and goosefoot as cyan. The per-class accuracy score is displayed near each colored circle (class label) for each considered feature.

Table 3 shows the classification results that were obtained with LGBM and QDA classifiers for each considered feature. Figure 6 and Figure 7 show the color-coded vegetation pixel classification of two test images using the LGBM classifier in weed detection and identification tasks, respectively.

Let us first compare the classification performance of reflectance against radiance features for the weed detection task. From the results that are given in Table 3, we can see that reflectance features estimated by illumination-based methods (rw, wa, ms, and dwd) provide better classification results than radiance features in terms of the average F1 and accuracy scores, whatever the classifier. The worst classification results are obtained with reflectance features that are estimated using the wn method. Training-based methods, such as wn, can provide an accurate reflectance estimation of scene objects whose optical properties are close to those of the training samples. In our case, the optical properties of vegetation are very different from that of the training ColorChecker patches. Thus, wn provides inaccurate reflectance estimations at vegetation pixels, which affects its classification performance. Figure 6 illustrates the satisfying $\bar{\mathrm{Accuracy}}$ and $\bar{F1}$ scores that are obtained thanks to the analysis of illumination-based reflectance features by LGBM. Indeed, this figure shows that weed is globally well detected by these methods.

For weed identification, the classification performances of all the features are degraded, because they provide weak performances on the goosefoot class (see Figure 7). This lack of generalization might be due to the high within-class dispersion (since we consider vegetation at various growth stages) and/or the physiological vegetation changes.

Let us now compare the classification performances of the reflectance features. The best overall classification results are obtained by our proposed rw method that performs well with both classifiers and reaches the highest average F1 and accuracy scores with LGBM for weed detection (85.4% and 86.1%, respectively). The wa method provides good classification results, better than those that were obtained by dwd, although the latter accounts for illumination variation during the frame acquisitions. The computation of illumination scaling factors to compensate for illumination variation may explain this poor performance, as well as the loss of spectral information (saturated reflectance values) in the near infra-red domain that is caused by illumination normalization (see Equation (A4)).

### 6.5. Experimental Conclusions

The experiments with this outdoor image database allow us to compare the performances of different reflectance features according to the estimation quality and pixel classification. The evaluation results are summarized by separately studying weed detection and identification. Table 4 and Table 5 show the rank ${\mathrm{Rank}}_{⋄,*}$ obtained by each reflectance estimation method ∗ according to each evaluation criterion ⋄ used in Table 1 and Table 3. The method with the lowest total rank is considered to be the best one, since it satisfies several criteria.

The total ranks of ms and wn methods are the highest ones, because they provide the worst results for either estimation quality (ms) or classification performance (wn). On the one hand, the dwd method that uses two reference devices to cope with illumination variation provides the second best total rank for weed detection (see Table 4). On the other hand, the wa method that uses one reference device, but assumes that illumination is constant, gives the second best total rank for weed identification (see Table 5). Our rw method, which row-wise analyzes one single reference device in order to take account of illumination variation, reaches the best total ranks for both weed detection and identification problems. These experiments suggest that rw-based reflectance features are relevant for weed identification under variable illumination conditions. Their performance should also be confirmed with other crop species, such as bean and wheat.

## 7. Conclusions

This paper first proposes an original image formation model of linescan multispectral cameras, like the Snapscan. It shows how illumination variation during the multispectral image acquisition by this device impacts the measured radiance that is provided by a Lambertian surface element. Our model is versatile and it can be adapted to model the outdoor image acquisition of several multispectral cameras, such as the HySpex VNIR-1800 [26] or the V-EOS Bragg-grating camera used in [16]. From this model, we propose a reflectance estimation method that copes with illumination variation. Because such varying conditions may affect the reflectance estimation quality, we estimate illumination at the frame level using a row-wise (rw) approach. We experimentally show that the rw method is more robust against illumination variation than the state-of-the-art methods. We also show that rw-based features are more discriminant to target outdoor supervised weed detection and identification, and they provide the best classification results. The accuracy of weed recognition systems and their robustness against illumination can be improved using reflectance features. This allows for precision spraying techniques to be considered in order to get rid of weeds using fewer quantities of chemicals. This study enables to make a step towards sustainable agriculture. As future work, segmentation will be extended to other plant species (such as wheat and bean) and growth stages. Spectral feature selection and texture features extraction will also be studied to improve the crop/weed identification performance. 

## Figures and Tables

**Table 1:** The reflectance estimation errors. Bold shows the best result and italics the second best one.

Method	Illumination-Based				Training-Based
	rw	wa	ms	dwd	wn
${\mathrm{MAE}}_{*}$(%)	4.315	5.883	14.670	**3.236**	*3.628*
${\Delta \theta }_{*}$ (rad)	**0.046**	**0.046**	0.309	*0.063*	*0.063*

**Table 2:** The number of learning and test pixels for crop/weed detection (left sub-column) and beet/thistle/goosefoot identification (right sub-column) (N=400,000 pixels in this experiment).

		$S^{\mathrm{learn}}$ (23 Images)			$S^{\mathrm{test}}$ (14 Images)		
**Class** $C_{i}$		**#Occurrences**	**# Learning Pixels** **per Occurrence**		**#Occurrences**	**#Test Pixels** **per Class**	
Crop	Beet	17	N/17		12	5,714,326	
Weed	Thistle Goosefoot	9 12	N/9 N/12	(N/2)/9 (N/2)/12	10 4	6,744,633	5,461,013 1,283,620

**Table 3:** Crop/weed detection and beet/thistle/goosefoot identification results with the QDA and LGBM classifiers. Bold shows best result and italics second best one.

			Radiance	Reflectance Features				
		Classifier	Feature	Illumination-Based				Training-Based
				**rw**	wa	ms	dwd	wn
Crop/weed detection	${\bar{\mathrm{Accuaracy}}}_{}$ (%)	QDA	71.6	76.0	73.3	**79.2**	*76.9*	48.1
		LGBM	77.0	**86.1**	*84.7*	81.8	83.4	71.0
	${\bar{F1}}_{}$ (%)	QDA	67.9	76.0	73.0	**77.8**	*76.5*	48.0
		LGBM	76.2	**85.4**	*84.4*	81.6	83.1	71.3
Beet/thistle/goosefoot identification	${\bar{\mathrm{Accuracy}}}_{}$ (%)	QDA	34.4	*45.1*	41.8	26.0	37.3	**47.8**
		LGBM	39.5	*49.7*	**50.3**	46.5	46.0	34.0
	${\bar{F1}}_{}$ (%)	QDA	34.3	**41.7**	*40.1*	25.4	33.7	27.0
		LGBM	39.7	**47.1**	*44.4*	41.4	42.5	32.9

**Table 4:** The ranking of reflectance estimation methods for crop/weed detection. Bold shows best result and italics second best one.

Evaluation Criterion			Method				
			rw	wa	ms	dwd	wn
*${\bar{\mathrm{MAE}}}_{*}$ (%)*			3	4	5	1	2
${\bar{\Delta \theta }}_{*}$ (rad)			1	1	5	3	3
Crop/weed detection	${\bar{\mathrm{Accuracy}}}_{*}$	QDA	3	4	1	2	5
		LGBM	1	2	4	3	5
	${\bar{F1}}_{*}$	QDA	3	4	1	2	5
		LGBM	1	2	4	3	5
$\sum_{⋄=1}^{6}{\mathrm{Rank}}_{⋄,*}$			**12**	17	20	*14*	25

**Table 5:** The ranking of reflectance estimation methods for crop/weed identification. Bold shows best result and italics second best one.

Evaluation Criterion			Method				
			rw	wa	ms	dwd	wn
*${\bar{\mathrm{MAE}}}_{*}$ (%)*			3	4	5	1	2
${\bar{\Delta \theta }}_{*}$ (rad)			1	1	5	3	3
Beet/thistle/goosefoot identification	${\bar{\mathrm{Accuracy}}}_{*}$	QDA	2	3	5	4	1
		LGBM	2	1	3	4	5
	${\bar{F1}}_{*}$	QDA	1	2	5	3	4
		LGBM	1	2	4	3	5
$\sum_{⋄=1}^{6}{\mathrm{Rank}}_{⋄,*}$			**10**	*13*	27	18	20

## Appendix A. Implementation of the Double White-Diffuser Method (dwd)

The double white diffuser (dwd) method [16] estimates reflectance thanks to three successive steps, that are adapted to our image contents as follows:

- Following Equation (14) but neglecting integration times and diffuse reflection factor, a coarse reflectance estimation is first computed as:
  (A1) $${\tilde{R}}_{p}^{b}=\frac{I_{p}^{b}}{I_{p}^{b}\left[\mathrm{WD}\right]}.$$
  Like in Section 3.1, this step aims to compensate the vignetting effect by a pixel-wise division of values $I_{p}^{b}\left[\mathrm{WD}\right]$ associated to the full-field white diffuser and $I_{p}^{b}$ associated to the scene. Note that a full-field white diffuser image $I^{\left(B\right)}\left[\mathrm{WD}\right]$ is acquired before each image acquisition.
- Because the illumination associated to $I^{\left(B\right)}\left[\mathrm{WD}\right]$ is different from that of the scene image $I^{\left(B\right)}$, $\tilde{R}$ is rescaled row-wise at each pixel *p* as:
  (A2) $${\tilde{R^{\prime }}}_{p}^{b}={\tilde{R}}_{p}^{b}\;\cdot \;\alpha_{y_{p}}^{b},$$
  where the illumination scaling factor $\alpha_{y_{p}}^{b}$ is computed at the row $y_{p}$ of *p* as:
  (A3) $$\alpha_{y_{p}}^{b}=\frac{{\bar{I}}_{\mathrm{WD},y_{p}}^{b}\left[\mathrm{WD}\right]}{{\bar{I}}_{\mathrm{WD},y_{p}}^{b}}.$$
  Each term in this equation is the average value over the row of *p* within the white diffuser subset WD in channel $I^{b}$ of either the full-field white diffuser image or the scene image.
- Finally, the values of ${\tilde{R}}^{\prime }$ are normalized channel-wise to provide the dwd reflectance estimation as:
  (A4) $${\hat{R}}_{\mathrm{dwd},p}^{b}={\tilde{R^{\prime }}}_{p}^{b}\;\cdot \;\frac{\rho_{\mathrm{WP}}^{b}}{\beta_{\mathrm{WP}}^{b}},$$
  where $\beta_{\mathrm{WP}}^{b}$ is the average value over the white patch subset WP in channel ${\tilde{R^{\prime }}}^{b}$, and $\rho_{\mathrm{WD}}^{b}$ is the diffuse reflection factor of the white patch for the spectral band centered at $\lambda^{b}$ measured by a spectroradiometer in laboratory.

The *B*-channel reflectance image ${\hat{R}}_{\mathrm{dwd}}^{\left(B\right)}$ undergoes spectral correction and negative value removal (see Equations (15) and (16)) to provide the final *K*-channel reflectance image ${\hat{R}}_{\mathrm{dwd}}^{\left(K\right)}$.

## References

[1] Wendel A., Underwood J. Self-supervised weed detection in vegetable crops using ground based hyperspectral imaging. Proceedings of the 2016 IEEE International Conference on Robotics and Automation (ICRA).

[2] Feyaerts F., van Gool L. (2001). Multi-spectral vision system for weed detection. Pattern Recognit. Lett..

[3] Lin F., Zhang D., Huang Y., Wang X., Chen X. (2017). Detection of Corn and Weed Species by the Combination of Spectral, Shape and Textural Features. Sustainability.

[4] Hagen N., Kudenov M.W. (2013). Review of snapshot spectral imaging technologies. Opt. Eng..

[5] Gat N. Imaging spectroscopy using tunable filters: A review. Proceedings of the SPIE: Wavelet Applications VII.

[6] Bianco G., Bruno F., Muzzupappa M. (2013). Multispectral data cube acquisition of aligned images for document analysis by means of a filter-wheel camera provided with focus control. J. Cult. Herit..

[7] Yoon S.C., Park B., Lawrence K.C., Windham W.R., Heitschmidt G.W. (2011). Line-scan hyperspectral imaging system for real-time inspection of poultry carcasses with fecal material and ingesta. Comput. Electron. Agric..

[8] Pichette J., Charle W., Lambrechts A. Fast and compact internal scanning CMOS-based hyperspectral camera: The Snapscan. Proceedings of the SPIE: Photonic Instrumentation Engineering IV.

[9] Shen H.L., Cai P.Q., Shao S.J., Xin J.H. (2007). Reflectance reconstruction for multispectral imaging by adaptive Wiener estimation. Opt. Express.

[10] Khan H.A., Thomas J.B., Hardeberg J.Y., Laligant O. (2019). Multispectral camera as spatio-spectrophotometer under uncontrolled illumination. Opt. Express.

[11] Heikkinen V., Lenz R., Jetsu T., Parkkinen J., Hauta-Kasari M., Jääskeläinen T. (2008). Evaluation and unification of some methods for estimating reflectance spectra from RGB images. J. Opt. Soc. Am..

[12] Bourgeon M.A., Paoli J.N., Jones G., Villette S., Gée C. (2016). Field radiometric calibration of a multispectral on-the-go sensor dedicated to the characterization of vineyard foliage. Comput. Electron. Agric..

[13] Del Pozo S., Rodríguez-Gonzálvez P., Hernández-López D., Felipe-García B. (2014). Vicarious Radiometric Calibration of Multispectral Camera on Board Unmanned Aerial System. Remote Sens..

[14] Uto K., Seki H., Saito G., Kosugi Y. (2013). Characterization of Rice Paddies by a UAV-Mounted Miniature Hyperspectral Sensor System. IEEE J-STARS.

[15] Khan H.A., Thomas J.B., Hardeberg J.Y., Laligant O. (2017). Illuminant estimation in multispectral imaging. J. Opt. Soc. Am..

[16] Eckhard J., Eckhard T., Valero E.M., Nieves J.L., Contreras E.G. (2015). Outdoor scene reflectance measurements using a Bragg-grating-based hyperspectral image. Appl. Opt..

[17] Zeng C., King D.J., Richardson M., Shan B. (2017). Fusion of Multispectral Imagery and Spectrometer Data in UAV Remote Sensing. Remote Sens..

[18] Goossens T., Geelen B., Pichette J., Lambrechts A., Van Hoof C. (2018). Finite aperture correction for spectral cameras with integrated thin film Fabry-Perot filters. Appl. Opt..

[19] Yu W. (2004). Practical anti-vignetting methods for digital cameras. IEEE Trans. Consum. Electron..

[20] Khan H.A., Mihoubi S., Mathon B., Thomas J.B., Hardeberg J.Y. (2018). HyTexiLa: High Resolution Visible and Near Infrared Hyperspectral Texture Images. Sensors.

[21] Amziane A., Losson O., Mathon B., Dumenil A., Macaire L. Frame-based reflectance estimation from multispectral images for weed identification in varying illumination conditions. Proceedings of the 2020 Tenth International Conference on Image Processing Theory, Tools and Applications (IPTA).

[22] Global Solar Irradiance in France. https://www.data.gouv.fr/fr/datasets/rayonnement-solaire-global-et-vitesse-du-vent-a-100-metres-tri-horaires-regionaux-depuis-janvier-2016/.

[23] Stigell P., Miyata K., Hauta-Kasari M. (2007). Wiener estimation method in estimating of spectral reflectance from RGB images. Pattern Recognit. Image Anal..

[24] Thenkabail P.S., Smith R.B., De Pauw E. (2002). Evaluation of Narrowband and Broadband Vegetation Indices for Determining Optimal Hyperspectral Wavebands for Agricultural Crop Characterization. Photogramm. Eng. Remote Sens..

[25] He H., Ma Y. (2013). Imbalanced Learning: Foundations, Algorithms, and Applications.

[26] HySpex VNIR-1800. https://www.hyspex.com/hyspex-products/hyspex-classic/hyspex-vnir-1800/.

